# Supplementary material for: Contractile forces in platelet aggregates under microfluidic shear gradients reflect platelet inhibition and bleeding risk
Source: Nat Commun. 2019 Mar 13;10:1204. doi: 10.1038/s41467-019-09150-9 (PMC6416331; doi:10.1038/s41467-019-09150-9)
Supplement: Supplementary file 1 — Supplementary Information [file 41467_2019_9150_MOESM1_ESM.pdf]

SUPPLEMENTARY INFORMATION

Contractile forces in platelet aggregates under microfluidic shear gradients reflect platelet inhibition and bleeding risk

Lucas H. Ting, Shirin Feghhi, Nikita Taparia, Annie O. Smith, Ari Karchin, Esther Lim, Alex St. John, Xu Wang, Tessa Rue, Nathan J. White, Nathan J. Sniadecki

<u>Supplementary Figures:</u>

Supplementary Figure 1. Processing steps for fabricating the microfluidic device with blocks and posts.

Supplementary Figure 2. Actin and P-selectin fluorescent staining of platelet aggregates formed on the block and post sensors.

Supplementary Figure 3. Flow cytometry of sheared blood through the microfluidic device.

Supplementary Figure 4. Immobilized protein is critical for formation of platelet aggregates under flow.

Supplementary Figure 5. Influence of shear rate on platelet forces and aggregate size.

Supplementary Figure 6. Platelet forces collected from healthy donors in either sodium citrate, lithium heparin, or into a syringe without anti-coagulant are equivalent.

Supplementary Figure 7. Representative phase contrast images of aggregates for inhibition studies of GPIb-IX-V and integrin $\alpha_{IIb}\beta_3$.

Supplementary Figure 8. Platelet forces are reduced in a dose-dependent manner with treatment of acetylsalicylic acid (ASA).

Supplementary Figure 9. Bench-top system for measuring platelet forces in a microfluidic device.

Supplementary Figure 10. Comparison of first-generation and second-generation designs of the microfluidic device.

Supplementary Figure 11. Platelet force and thromboelastography data for an in vitro model of trauma induced coagulopathy.

Supplementary Figure 12. Hemodilution of whole blood with Ringer's solution.

Supplementary Figure 13. Platelet forces versus platelet count for trauma patients.

Supplementary Figure 14. Platelet forces versus fibrinogen concentration for trauma patients.

Supplementary Tables:

Supplementary Table 1. Cardiology patient medication profiles. Enrollment was dependent on taking aspirin regularly.

Supplementary Table 2. Demographics, vital signs, laboratory values, and outcomes for transfused and non-transfused Emergency Department trauma patients.

Supplementary Table 3. Mean results for thromboelastometry and impedance aggregometry results for non-transfused and transfused subjects.

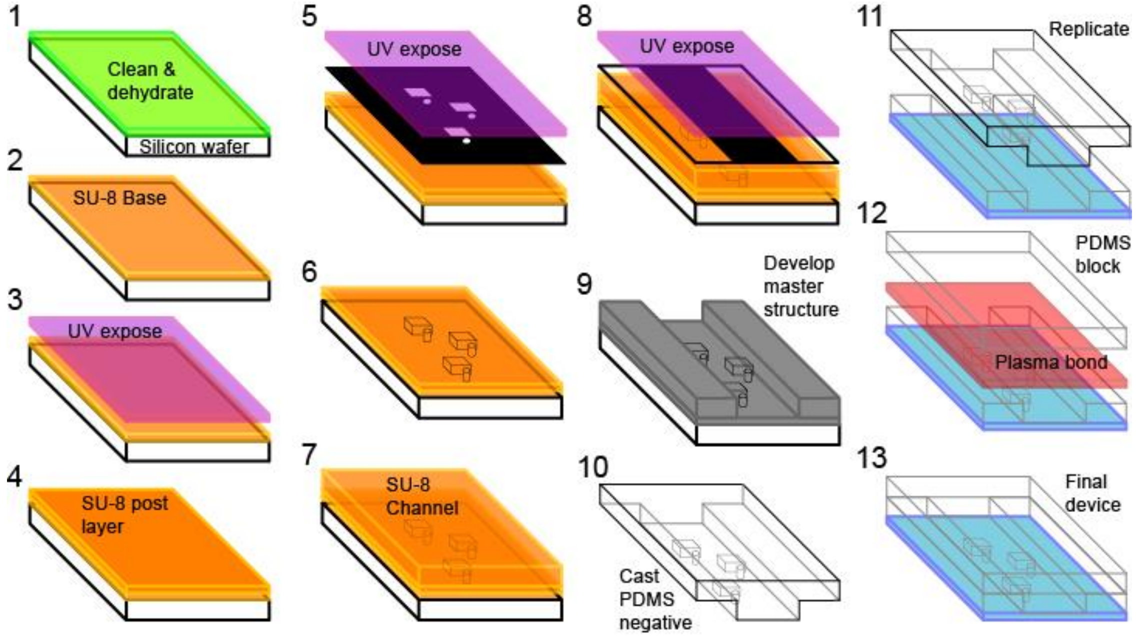

Supplementary Figure 1. Processing steps for fabricating the microfluidic device with blocks and posts. First, a silicon wafer was cleaned using a series of washes (acetone, isopropyl alcohol, and deionized water) and then held at 200 °C to fully clean and dry its surface of impurities (step 1). Next, a negative type photoresist (SU-8 2000 series, Microchem) was spin-coated to form a base layer (step 2). Next, a series of UV exposures (steps 3, 5, and 8) and spin coatings (steps 4, 6, and 7) were performed to form features in the photoresist. These steps resulted in a master structure (step 9). Prior to using the master, we treated it with vapors of fluorosilane (T2492, United Chemical Technologies) under vacuum in a desiccator. Several negative molds were made from the master by mixing PDMS at a 10:1 ratio with a curing agent, pouring it onto the master structure, and then curing for 20 minutes at 110 °C. The mold was treated for 90 seconds with plasma (Plasma Prep II, Structure Probe, Inc.) and then treated with vapors of fluorosilane under vacuum in a desiccator (step 10). The negative mold was used for casting the PDMS replicates that formed the microfluidic devices (step 11). Here, PDMS was mixed at a 10:1 ratio and poured onto the mold. The mold with PDMS was set onto a #0 glass coverslip and cured at 110 °C for 24 hours. The coverslip with cured PDMS was peeled away from the mold, leaving an open-topped microchannel with blocks and post sensors on the coverslip. The top layer for the microchannels was made from a thin film of PDMS that was cut to match the length and width of the microfluidic device. Holes were punched into the top layer for the inlet and outlet ports. The top PDMS layer of the microchannel was attached by treating the PDMS microchannels and top layer with plasma for 90 seconds and pressing together to create a strong, water-tight bond between the two surfaces (step 12), resulting in the final form of the microfluidic device (step 13).

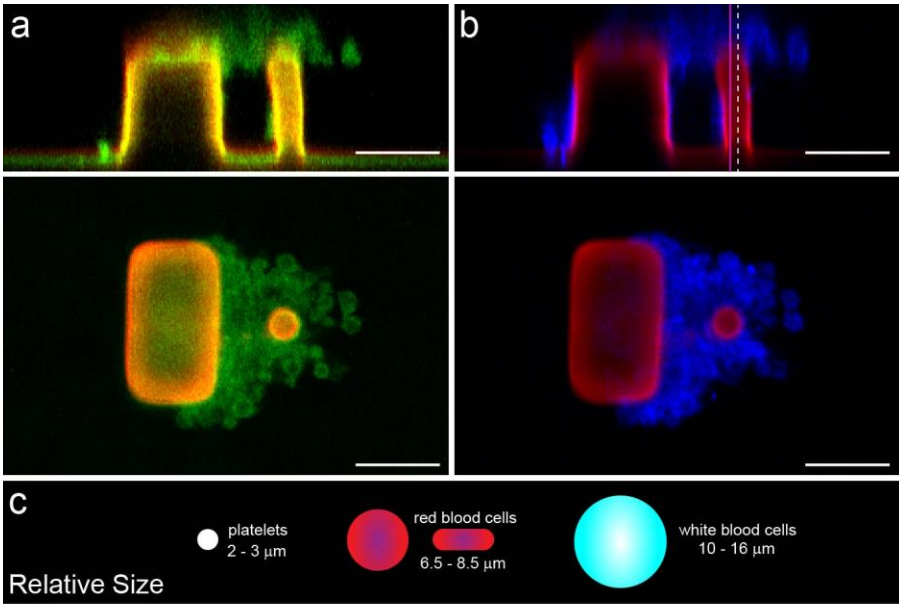

Supplementary Figure 2. Actin and P-selectin fluorescent staining of platelet aggregates formed on the block and post sensors. Representative images of samples labeled for both (**a**) actin and (**b**) P-selectin confirm the presence of platelets and absence of other cell types. (**c**) For comparison, the relative size of a platelet, RBC, and WBC is shown in accordance with the scale of panels A and B. To obtain these images, samples of whole blood was flowed through the microfluidic device at 8000 s^{-1} and then fixed using 4% paraformaldehyde (Electron Microscopy Supplies) in DI water for 2 hours. Tyrode buffer was used to flush out the fixing solution. A solution 0.2% Triton X-100 (Sigma) was added for 10 minutes to permeabilize the clot structure. The sample was flushed with PBS buffer twice. A solution of 10% goat serum (Invitrogen) was added for blocking, and then 1:200 rabbit P-selectin (Thermo Fisher, Cat. No. 710281) in 10% goat serum was injected and allowed to incubate for 30 minutes. It was then flushed with PBS and 10% goat serum was added for 30 minutes. A secondary stain of 1:400 phalloidin Alexa Fluor 488 and 1:200 goat anti-rabbit Alexa Fluor 647 (Thermo Fisher, Cat. No. A-21244) was added and incubated for 30 minutes. After flushing with PBS and water, Fluoromount-G was injected into the microchannel to preserve the staining of the sample for microscopy. Confocal images were taken on a Zeiss laser scanning microscope 510 META. Scale bar, 10 μm.

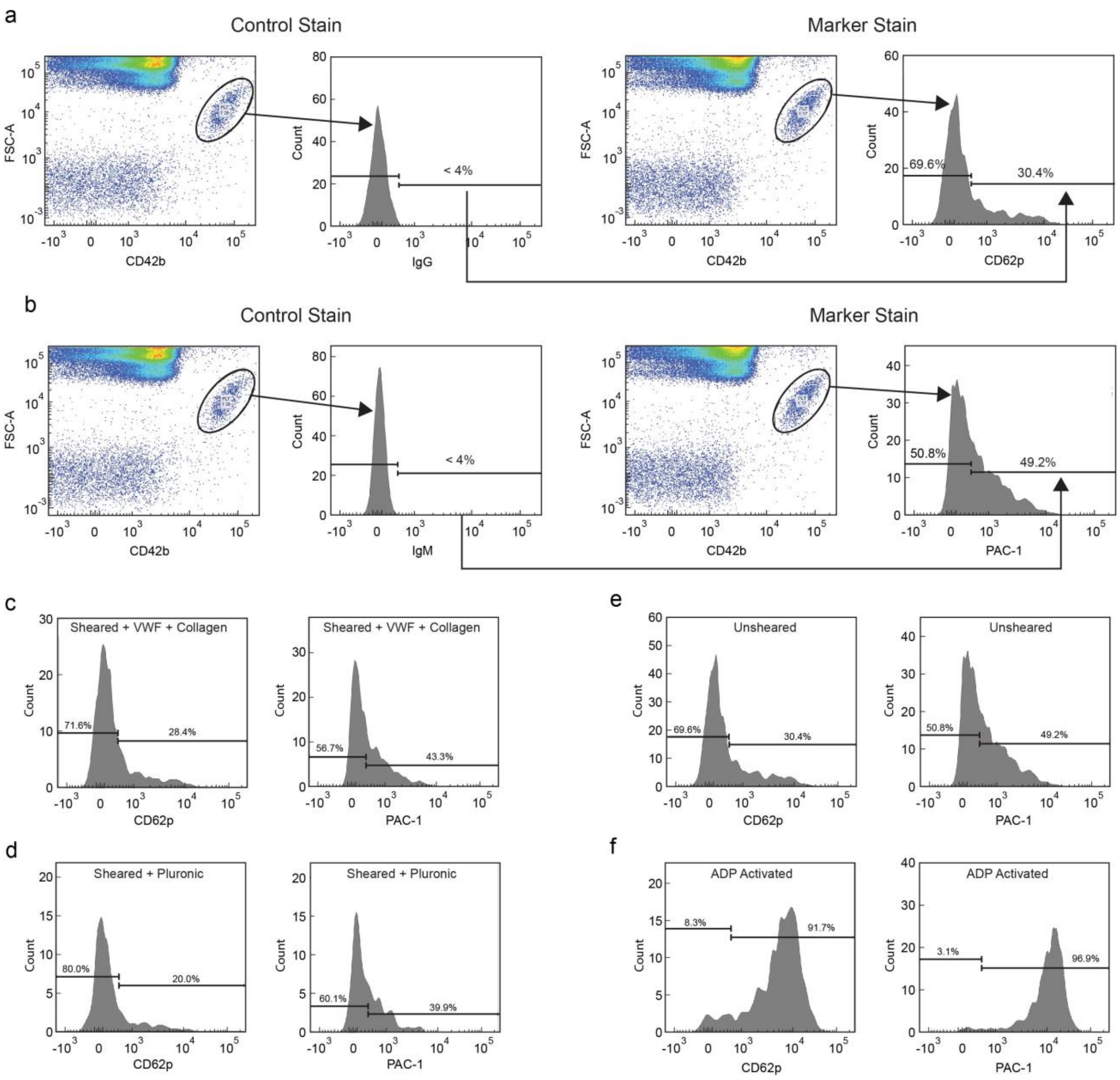

Supplementary Figure 3. **Flow cytometry of sheared blood through the microfluidic device.** We evaluated the expression levels of P-selectin and activated $\alpha_{IIb}\beta_3$ in platelets of a blood sample that was sheared through the microfluidic devices at 8000 s^{-1}. Gating strategies were used to determine the amount of (**a**) P-selectin via CD62p antibody binding and (**b**) activated $\alpha_{IIb}\beta_3$ via PAC-1 binding above the background of nonspecific isotype binding. Platelets in a blood sample were stained with CD42b-APC monoclonal antibody (BD Biosciences, Cat. No. 170429) at 7% dilution from stock and CD62p-PE or PAC-1-FITC monoclonal antibodies (BD Biosciences, Cat. No. 555524 and 340507) at 13% dilution from stock. Non-specific binding was detected and subtracted by staining separately with isotype control antibody with the same fluorophore, IgG-PE and IgM-FITC (BD Biosciences, Cat. No. 559320 and 553474) at 13% and 7% dilution, respectively. Following labeling, platelets were fixed in 1% paraformaldehyde and then measured on a BD LSR II flow cytometer (BD Biosciences). For each sample, a gate was drawn that was positive for less than 4% of platelets

stained with IgG or IgM antibodies, which defined the limit of nonspecific binding. For testing, tThe surfaces of the microchannels, blocks, and posts were coated with either (**c**) collagen and VWF to promote platelet adhesion or (**d**) 0.2% Pluronic F-127 (Sigma) to prevent adhesion. (**e**) An unsheared blood sample served as a negative control and (**f**) an unsheared blood sampled activated with 15 µM adenosine diphosphate (ADP) at the time of staining served as a positive control. These results show that sheared platelets have similar levels of P-selectin and activated $\alpha_{IIb}\beta_3$ as unsheared blood, indicating that they have minimal levels of activation after passing through the microfluidic devices.

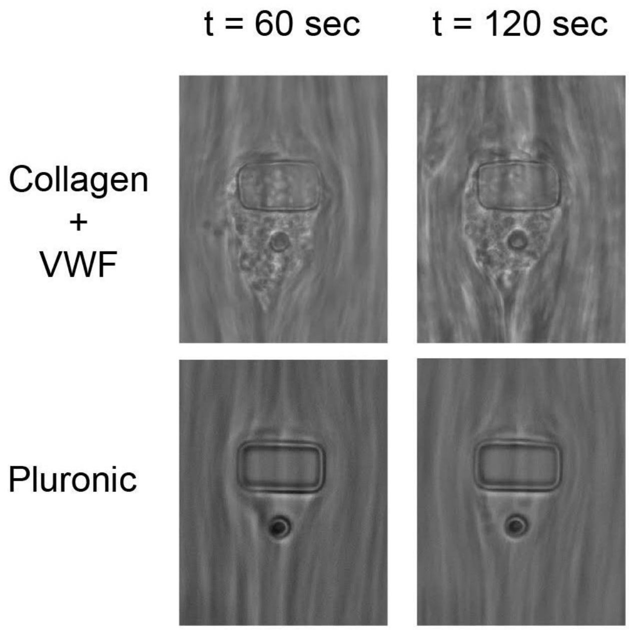

Supplementary Figure 4. Immobilized protein is critical for formation of platelet aggregates under flow. Representative images of platelet aggregates formed on a block and post coated with collagen and von Willebrand factors (VWF) to enable platelet adhesion under flow or coated with 0.2% Pluronic F-127 to block the adsorption of soluble matrix proteins onto the surface the block and post and thereby prevent platelet adhesion.

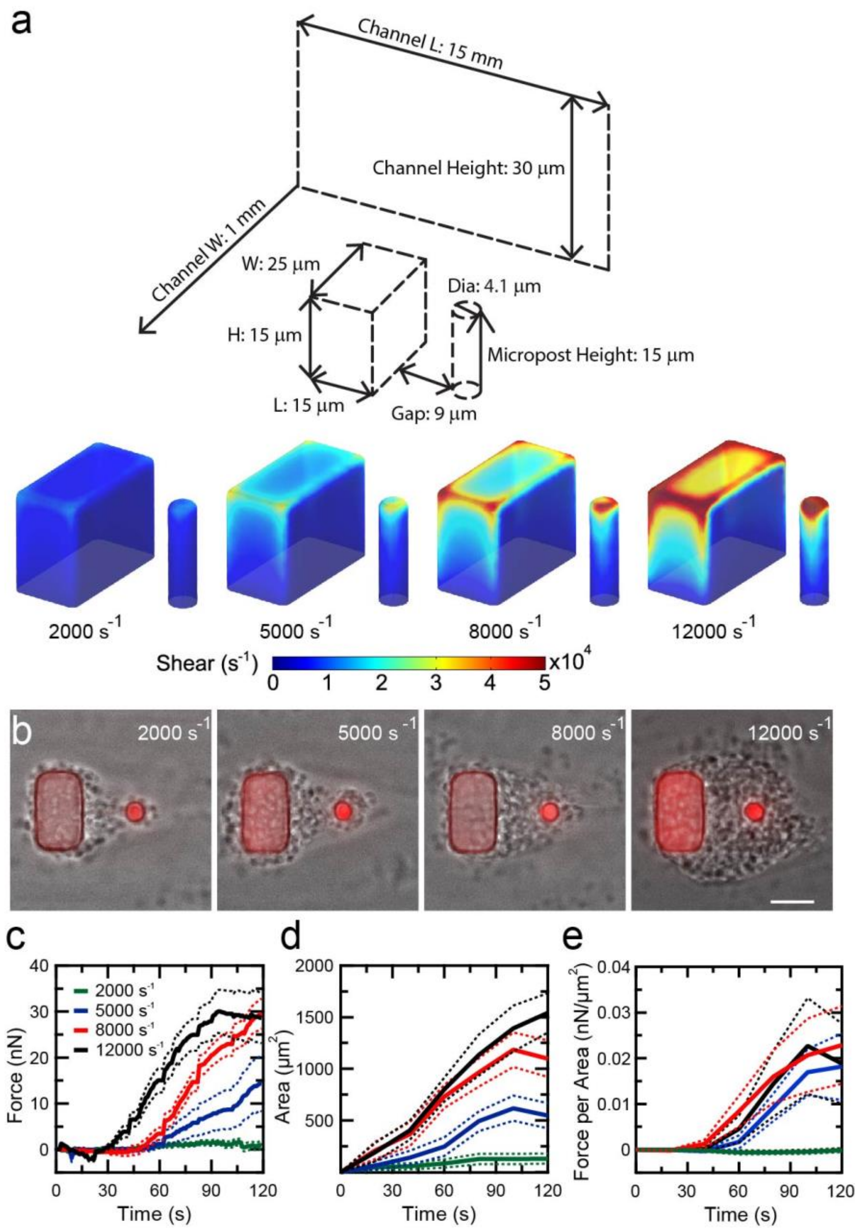

Supplementary Figure 5. Influence of shear rate on platelet forces and aggregate size. To investigate whether the generation of platelet forces is dependent on shear rate, blood samples were tested in the microchannels at a wall shear rate of 2000, 5000, 8000, or 12000 s^{-1}. (**a**) Computational fluid dynamics simulations with the dimensions of the block, micropost, and microchannel show local regions of high shear increase in proportion to the wall shear rate. The highest and lowest magnitudes for the wall shear gradients at the block were 5.74E6 s^{-1} mm^{-1} and -2.15E6 s^{-1} mm^{-1} for a wall shear rate of 8000 s^{-1}. (**b**) Representative images of platelet aggregates formed after 120 seconds of flow demonstrate that larger structures form at higher shear rates. Scale bar, 10 µm. (**c**) Forces and (**d**) projected area of the aggregates were found to depend on wall shear rate. We noted that at higher shear rates, there was a greater rate of accumulation of platelets onto the outmost portion of the aggregates. We attributed the increased rate of accumulation to the flow rate because it affects the number of platelets that transit over the block and posts. (**e**) To account for the different rates of accumulation, we

divided the force of an aggregate by its projected area, *i.e.* force per area. We find that for wall shear rates at 8000 s^{-1} and higher, there is not a significant effect on force per area. Solid lines represent averages from all experiments and dashed lines represent standard error of the mean. Data in graphs are from N≥5 independent experiments where N≥4 block-posts were analyzed per experiment.

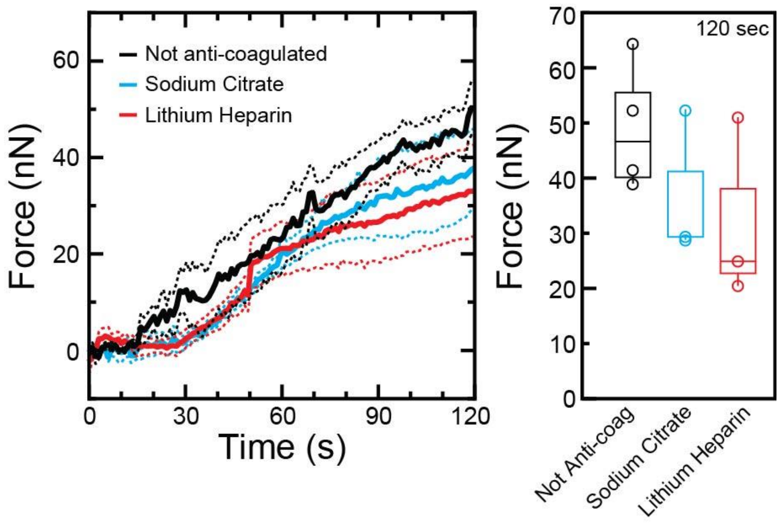

Supplementary Figure 6. Platelet forces collected from healthy donors in either sodium citrate, lithium heparin, or into a syringe without anti-coagulant are equivalent. Whole blood was collected in blood collection tubes with sodium citrate, lithium heparin, or directly into an empty 1 mL syringe and tested within 5 minutes of draw. The onset of force was delayed in citrated and heparinized samples as compared to samples that were not anti-coagulated. The force at 120 seconds were lower for the citrated and heparinized samples as compared to the not anti-coagulated samples, but there was not a statistical difference between these measurements. Solid lines represent averages from all experiments and dashed lines represent standard error of the mean. Body of the box plots represent first and third quartiles. Center lines denotes the median. Whiskers extent from the quartiles to the last data point within 1.5× interquartile range, with outliers beyond. Data shown is from at least 3 donors per condition. There was no statistical difference between data measured by ANOVA with a Tukey post-hoc test.

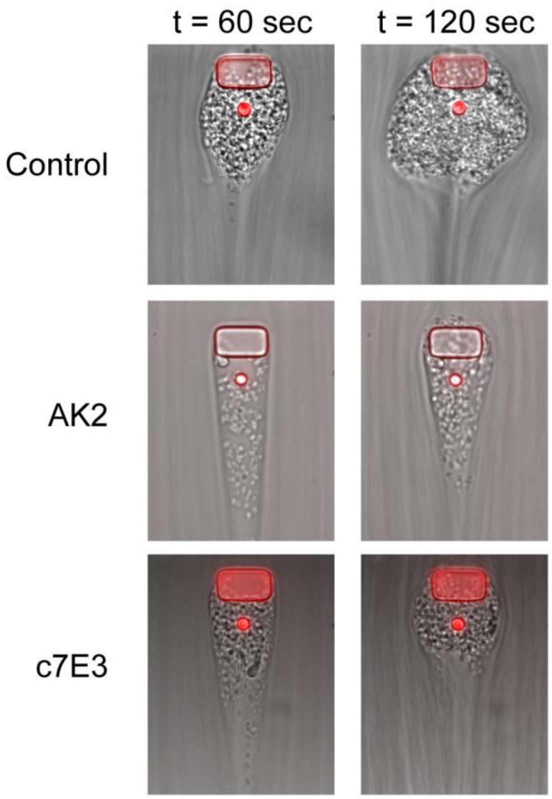

Supplementary Figure 7. Representative phase contrast images of aggregates for inhibition studies of GPIb-IX-V and integrin $\alpha_{IIb}\beta_3$. Whole blood was incubated with AK2 (anti-GPIb) or c7E3 (anti-αIIbβ3) antibodies for 20 minutes and then flowed across the array of block and post sensors at 8000 s^{-1}. Platelets formed smaller aggregates with the antibody treatments as compared to control. All sensors within an image are analyzed and averaged together to obtain one data point per donor.

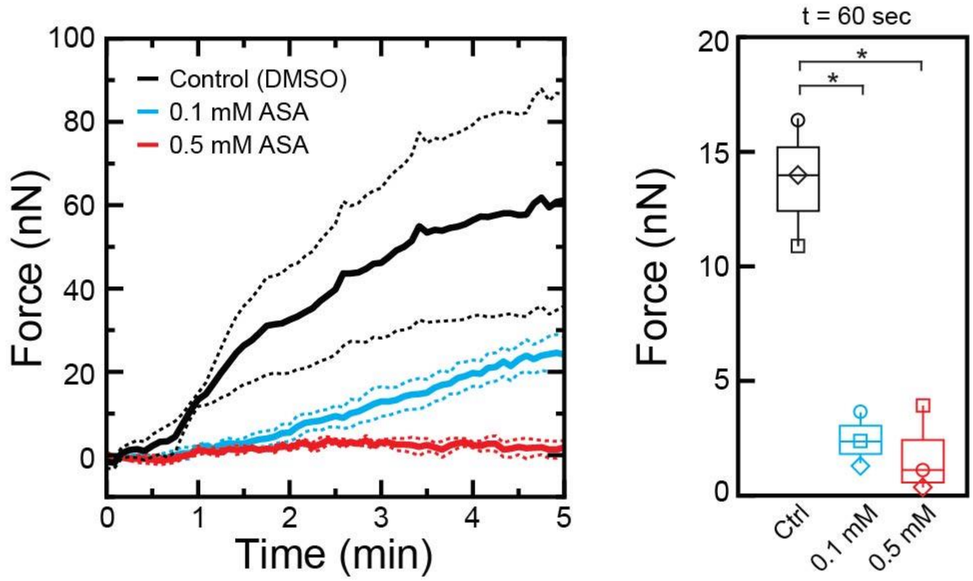

Supplementary Figure 8. Platelet forces are reduced in a dose-dependent manner with treatment of acetylsalicylic acid (ASA). Addition of 0.1 mM ASA is equivalent to an 81 mg dose in an average adult, while 0.5 mM ASA is higher than a 325 mg dose in an average adult. Dotted lines are standard error of the mean. Significance was found between the ASA treated samples and the control at 60 seconds of shear flow. Solid lines represent averages from all experiments and dashed lines represent standard error of the mean. Body of the box plots represent first and third quartiles. Center lines denotes the median. Whiskers extent from the quartiles to the last data point within 1.5× interquartile range, with outliers beyond. Asterisks denote $p < 0.05$, ANOVA with a Tukey post-hoc test. Data shown is from three donors.

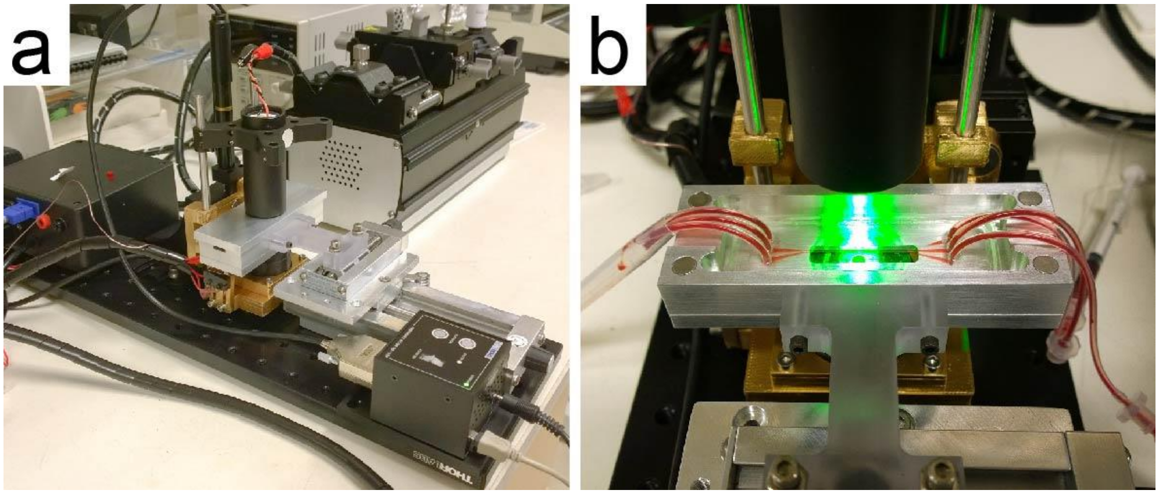

Supplementary Figure 9. Bench-top system for measuring platelet forces in a microfluidic device. (**a**) A custom-built device was used to measure platelet forces in blood samples from trauma patients. It consists of a heated (37 °C) aluminum chamber that is mounted on an x-y stage and is sized to fit a microfluidic card. A CMOS camera is attached to a motorized z-axis controller for adjusting its focus to the microfluidic card. The movement of block and post sensors within the microfluidic card is recorded under fluorescence microscopy. Specifically, a white-light LED is mounted above the microfluidic card and is coupled with an excitation filter (542 ± 10 nm) and an aspheric condenser lens. An emission filter (620 ± 26 nm) is position between the lens of the camera and its CMOS sensor. A syringe pump shown in the background holds a syringe and controls the rate of blood flowing into the microfluidic card. (**b**) A close-up view of the aluminum chamber showing the heated interior and microfluidic card. Blood enters from the right side of the device through silicone tubes, flows through an illuminated area where the block and post sensors are fluorescently imaged, and then exits the card into a waste receptacle seen on the left. The lens of the camera is seen through the slot in the center of the heated chamber.

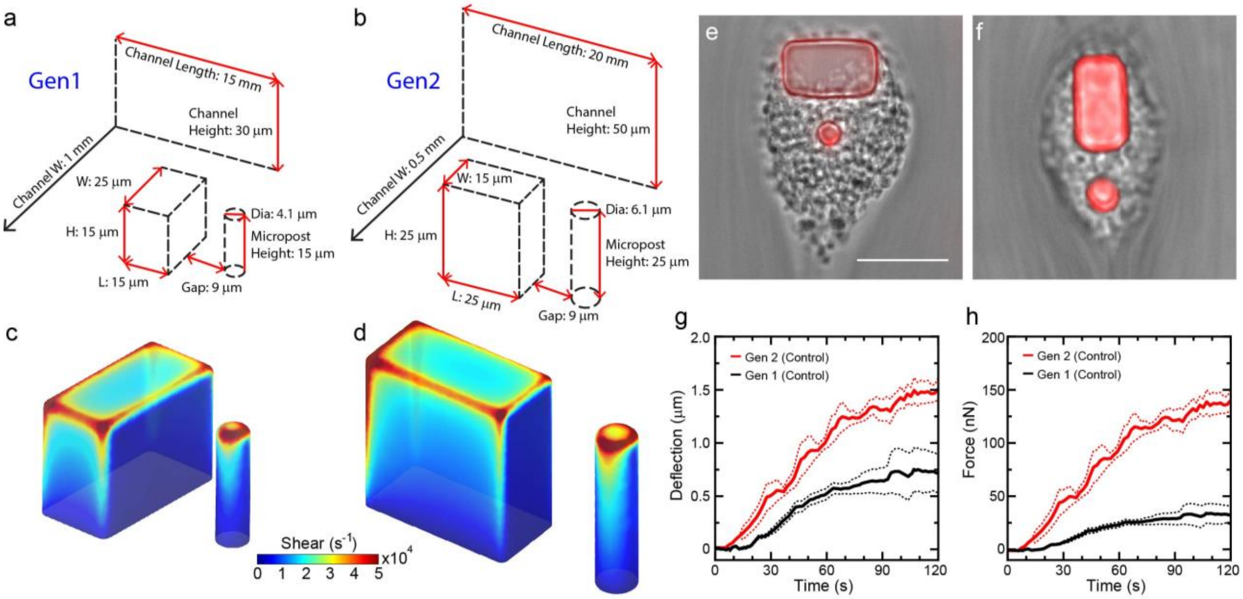

Supplementary Figure 10. Comparison of first-generation and second-generation designs of the microfluidic device. (**a-b**) We sought to increase the visible range of deflection of the posts to improve the resolution of force by image analysis with the bench-top system (Fig. S8), so we increased the height and diameter of the post in the second-generation design (Gen 2), while keeping its bending stiffness approximately equivalent to the first-generation design (Gen 1), *i.e*. 46.9 *vs*. 49.6 nN/µm. The height of the block was increased to match with the height of the post and its position was rotated by 90° to increase its second moment of area to ensure that the block was a rigid structure. The height of the microchannel was increased to accommodate the taller posts while the width was reduced to enable the camera on the bench-top system to monitor three microchannels simultaneously (**c-d**) Guided by the data on shear rate shown in Supplementary Figure 4, we doubled the shear rate to 16,000 s^{-1} for the first 15 seconds of the test so platelets accumulated faster on the sensors and thereby imparted a greater force on the post. The shear rate was then decreased to 500 s^{-1} to reduce the rate of new platelets accumulating on the sensors. CFD simulations show a similar range of shear rates across the block and post sensors for Gen 1 versus Gen 2. In this configuration, the shear gradients at a block in the Gen 2 design were -2.76×10^6 s^{-1} mm^{-1} and 7.78×10^6 s^{-1} mm^{-1}, which were similar to those in the Gen 1 design. By increasing the height of the post and the shear rate, (**e-f**) similar sized aggregate develops on the block and post for blood samples from healthy donors, but (**g**) the deflection of the post is larger, as well as (**h**) the forces measured. Solid lines represent averages from all experiments and dashed lines represent standard error of the mean. Data shown is from 3 donors for Gen 1 and 10 donors for Gen 2. Scale bar, 25 µm.

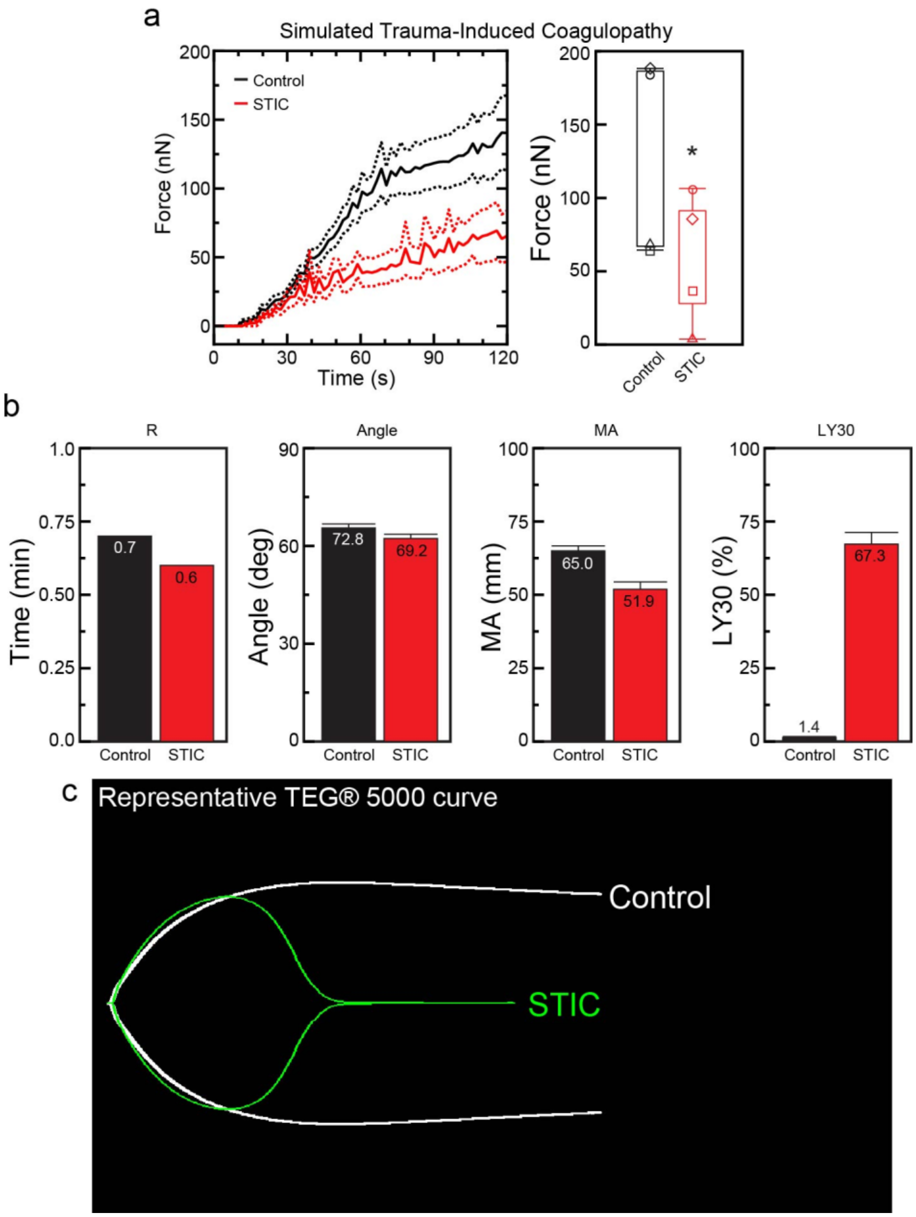

Supplementary Figure 11. Platelet force and thromboelastography data for an *in vitro* model of trauma induced coagulopathy. Citrated blood from healthy donors was split into two portions and one portion was diluted with 15% Ringer's solution and 225 ng mL^{-1} tissue plasminogen activator (EMD Millipore) and 45 pmol L^{-1} tissue factor (Haemotologic Technologies) for 20 minutes to simulate the coagulation response seen in trauma patients with TIC (STIC). The other portion was left untreated as a control. The samples were measured for (**a**) platelet forces and (**b**) reaction time, rate of clot formation (angle), clot strength (MA), and clot lysis (LY30). (**c**) Representative thromboelastography curve produced on a TEG®-5000 (Haemonetics) for the STIC and control conditions is also shown for reference. Data shown is from five donors. Solid lines represent averages from all experiments and dashed lines represent standard error of the mean. Body of the box plots represent first and third quartiles. Center lines denotes the median. Whiskers extent from the quartiles to the last data point within 1.5× interquartile range, with outliers beyond. Data points represent results from a single donor. Error bars denote standard error of the mean. Asterisks denote $p < 0.05$, paired T-test.

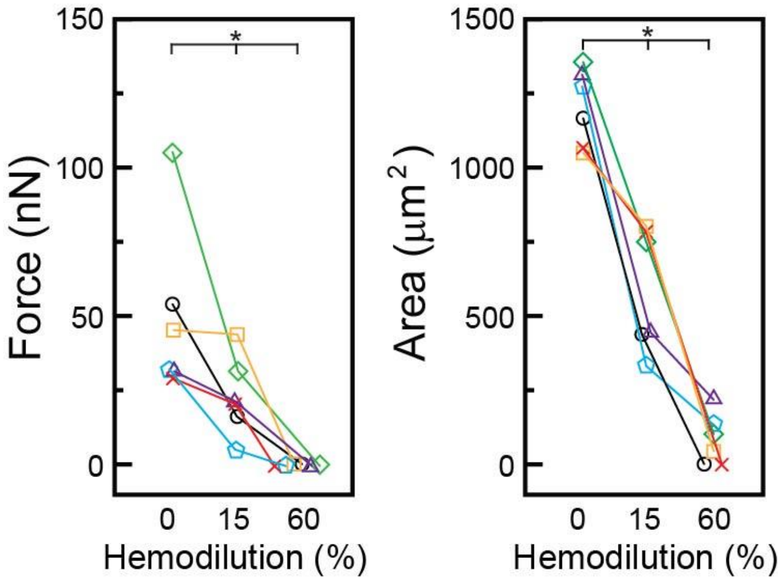

Supplementary Figure 12. Hemodilution of whole blood with Ringer's solution. Blood samples from six healthy donors were mixed with 1X Ringer's solution to 0%, 15%, and 60% dilution before being sheared across the Gen 2 device. Results after 2 minutes of testing indicate that there is a difference in force and area at 15% and 60% dilution (one-way ANOVA, F=11.903, DF=2, p=0.0008). Each marker shape represents results from each individual donor. Asterisks denote $p < 0.01$ between all three comparison groups with Tukey post-hoc test.

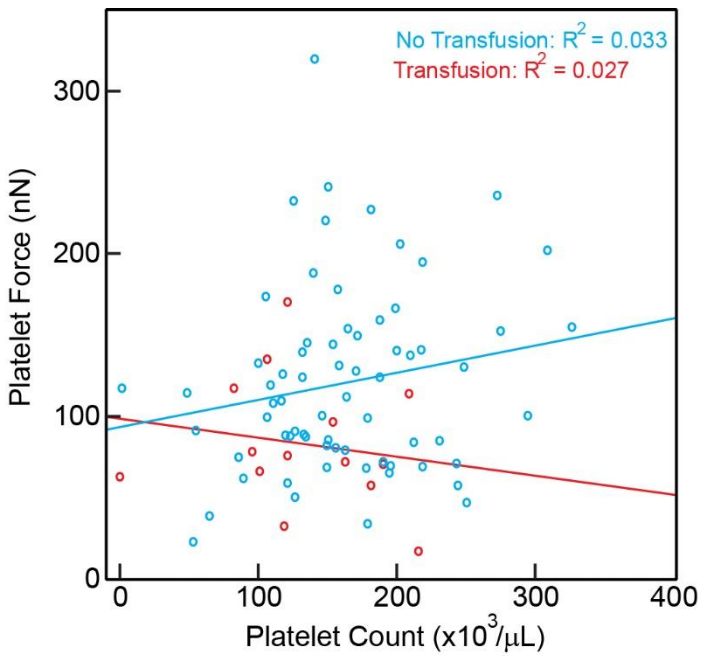

Supplementary Figure 13. Platelet forces versus platelet count for trauma patients. Forces and platelet counts were measured for emergency room trauma patients. R-squared values indicate that there was not a relationship between platelet forces and platelet count. High platelet forces and very low platelet forces were both observed in trauma patients with normal platelet counts (150,000-400,000 per µL).

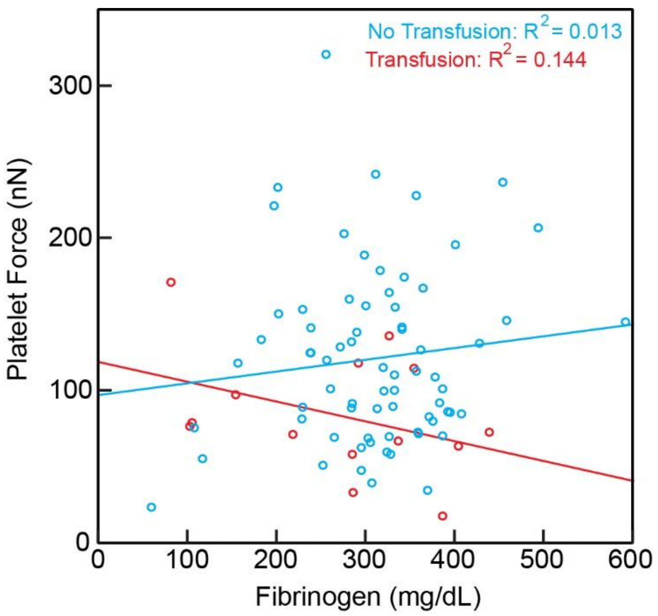

Supplementary Figure 14. Platelet forces versus fibrinogen concentration for trauma patients. Forces and fibrinogen concentration were measured for emergency room trauma patients. R-squared values indicate that there was not a relationship between platelet forces and fibrinogen concentration.

Supplementary Table 1. Cardiology patient medication profiles. Enrollment was dependent on taking aspirin regularly.

Patient	Aspirin (mg/day)	Additional medication (mg/day)
#1	aspirin: 81	lisinopril: 5
#2	aspirin: 75	warfarin: 17.5, carvedilol: 6.25, torsemide: 100, amiodarone: 200
#3	aspirin: 325	warfarin: 11, lisinopril: 2.5, metoprolol: 25, digoxin: 0.125, toresmide: 20
#4	aspirin: 81	warfarin: 5, torsemide: 150, carvedilol: 12.5, simvastatin: 20
#5	aspirin: 81	lisinopril: 2.5, toresmide: 20, pravastatin: 10, amlodipine: 10
#6	aspirin: 325	warfarin: 17.5, metoprolol: 25, digoxin: 0.125, lisinopril: 20
#7	aspirin: 81	warfarin: 7.5, metoprolol: 25, digoxin: 0.125, simvastatin: 30, ... amiodarone: 200, losartan: 50, amlodipine: 10

Supplementary Table 2. Demographics, vital signs, laboratory values, and outcomes for transfused and non-transfused Emergency Department trauma patients.

	No Transfusion			Transfusion			
**Demographics and Outcomes**	**N**	**Mean**	**Std Dev**	**N**	**Mean**	**Std Dev**	**P value**
Age	80	**47.3**	16.9	12	**58.1**	24.4	0.16
Sex (% Male)		**75%**			**58%**		0.24*
Mechanism (% blunt)		**85%**			**83%**		0.88*
Died in Hospital N (%)		**3(3.8%)**			**4(33.3%)**		0.003*
Injury Severity Score	80	**14.0**	10.0	12	**33.2**	16.0	0.002
**Prehospital Intravenous Fluids (ml)**	57	878.1	700.4	13	1,253.9	910.7	0.18
**Emergency Department Vital Signs**							
Systolic Blood Pressure (mmHg)	80	**138.9**	23.9	12	**94.2**	38.0	0.002
Pulse (beats per min)	80	**89.3**	20.5	11	**99.9**	33.2	0.32
Respiratory Rate (breaths per min)	80	**17.4**	3.8	11	**16.1**	6.3	0.52
Temperature (°C)	60	**36.5**	0.5	7	**36.2**	0.5	0.18
**Hematology***							
White Blood Cell Count (cells mcL^{-1})	91	**10.9**	4.6	15	**12.0**	7.1	0.58
Hemoglobin (g dL^{-1})	93	**13.6**	1.5	17	**10.8**	3.7	0.006
Hematocrit (%)	93	**40.3**	4.0	17	**36.2**	12.1	0.19
Platelet count (cells mcL^{-1})	93	**230.5**	52.6	16	**193.2**	95.8	0.15
**Coagulation***							
Prothrombin Time (sec)	90	**14.2**	2.3	15	**17.7**	6.4	0.058
Activated Partial Thromboplastin Time (sec)	90	**31.2**	4.9	15	**43.8**	17.9	0.02
Fibrinogen (mg dL^{-1})	90	**308.2**	87.6	15	**267.3**	115.4	0.21
**Arterial Blood Gas***							
pH	46	**7.4**	0.1	16	**7.3**	0.1	0.67
PCO_2 (mmHg)	46	**41.2**	11.6	16	**40.8**	9.6	0.88
PO_2 (mmHg)	46	**237.5**	156.8	16	**97.3**	65.1	<0.001
Base deficit (meq L^{-1})	46	**-2.6**	2.7	16	**-4.7**	5.1	0.14
Hemoglobin Oxygen Saturation (%)	28	**90.6**	19.0	13	**79.6**	27.0	0.2
Lactate (mmol L^{-1})	47	**2.9**	2.0	16	**4.3**	3.1	0.1
**Blood Products Transfused in 24 hr (units)**							
Packed Red Blood Cells	93	**0**	0	17	**4.2**	4.4	0.001
Platelet Concentrates	93	**0**	0	17	**1.1**	1.2	0.003
Cryoprecipitate	93	**0**	0	17	**0.5**	0.9	0.02
Fresh Frozen Plasma	93	**0**	0	17	**3.1**	5.1	0.02

[#] First result recorded in the Emergency Department, P values are result of unequal variance two-sided T test or Chi Square Likelihood Ratio (*).

Supplementary Table 3. Statistical tests for thromboelastometry and aggregometry measurements for trauma patients.

	No 24hr Transfusion		24hr Transfusion		T-Test
**Test**	Mean	Std Dev	Mean	Std Dev	P value
**EXTEM Whole Blood (fibrin+platelets)**					
Clotting Time (sec)	**60.4**	13.0	**407.3**	1334.4	0.32
Clot Formation Time (sec)	**92.0**	31.0	**101.9**	48.4	0.45
Alpha Angle (°)	**72.2**	6.6	**75.6**	46.0	0.77
Clot Amplitude at 10 min (mm)	**54.4**	7.5	**47.8**	16.5	0.13
Maximal Clot Firmness (mm)	**63.1**	6.5	**56.3**	18.1	0.17
Lysis Index at 30 min (% of maximal firmness)	**99.2**	7.1	**99.6**	1.6	0.65
**EXTEM Plasma (fibrin only)**					
Clotting Time (sec)	**57.3**	12.7	**396.0**	1337.4	0.33
Clot Formation Time (sec)	**397.9**	696.5	**308.2**	236.9	0.43
Alpha Angle (°)	**78.3**	6.9	**71.4**	22.8	0.32
Clot Amplitude at 10 min (mm)	**24.2**	7.1	**18.5**	10.0	0.04
Maximal Clot Firmness (mm)	**27.0**	9.4	**23.2**	17.9	0.41
Lysis Index at 30 min (% of maximal firmness)	**99.5**	1.6	**93.7**	22.1	0.34
**Impedance Platelet Aggregometry (area under the aggregation curve)**					
ADP Activation	**53.9**	26.9	**39.7**	28.5	0.09
Arachidonic Acid Activation	**42.2**	24.8	**28.9**	24.0	0.06
Collagen Activation	**39.0**	23.8	**31.5**	26.1	0.32
TRAP Activation	**96.8**	32.7	**87.1**	38.9	0.37
Ristocetin Activation	**41.8**	29.8	**32.3**	32.6	0.30

EXTEM = rotational thromboelastometry (ROTEM) with extrinsic pathway activation
ADP = adenosine diphosphate
TRAP = thrombin receptor activator peptide.
P values are result of unequal variance two-sided T test.
