# Supplementary material for: Contractile forces in platelet aggregates under microfluidic shear gradients reflect platelet inhibition and bleeding risk
Source: Nat Commun. 2019 Mar 13;10:1204. doi: 10.1038/s41467-019-09150-9 (PMC6416331; doi:10.1038/s41467-019-09150-9)
Supplement: Supplementary file 3 — Description of Additional Supplementary Files [file 41467_2019_9150_MOESM3_ESM.docx]

**Description of Supplementary Files**

**File Name:** Supplementary Movie 1.

**Description:** Video of whole blood flowing by a microscale block and post.

**File Name:** Supplementary Movie 2.

**Description:** Video of intracellular calcium concentration within a platelet aggregate that forms on a block and post.
