# Supplementary material for: Contractile forces in platelet aggregates under microfluidic shear gradients reflect platelet inhibition and bleeding risk
Source: Nat Commun. 2019 Mar 13;10:1204. doi: 10.1038/s41467-019-09150-9 (PMC6416331; doi:10.1038/s41467-019-09150-9)
Supplement: Supplementary file 6 — Reporting Summary [file 41467_2019_9150_MOESM6_ESM.pdf]

nature research

Corresponding author(s): Nathan Sniadecki, Nathan White

Last updated by author(s): Jan 11, 2019

Reporting Summary

Nature Research wishes to improve the reproducibility of the work that we publish. This form provides structure for consistency and transparency in reporting. For further information on Nature Research policies, see Authors & Referees and the Editorial Policy Checklist.

Statistics

For all statistical analyses, confirm that the following items are present in the figure legend, table legend, main text, or Methods section.

n/a	Confirmed	
☐	☒	The exact sample size (*n*) for each experimental group/condition, given as a discrete number and unit of measurement
☐	☒	A statement on whether measurements were taken from distinct samples or whether the same sample was measured repeatedly
☐	☒	The statistical test(s) used AND whether they are one- or two-sided<br>*Only common tests should be described solely by name; describe more complex techniques in the Methods section.*
☒	☐	A description of all covariates tested
☐	☒	A description of any assumptions or corrections, such as tests of normality and adjustment for multiple comparisons
☐	☒	A full description of the statistical parameters including central tendency (e.g. means) or other basic estimates (e.g. regression coefficient) AND variation (e.g. standard deviation) or associated estimates of uncertainty (e.g. confidence intervals)
☐	☒	For null hypothesis testing, the test statistic (e.g. *F*, *t*, *r*) with confidence intervals, effect sizes, degrees of freedom and *P* value noted<br>*Give P values as exact values whenever suitable.*
☒	☐	For Bayesian analysis, information on the choice of priors and Markov chain Monte Carlo settings
☒	☐	For hierarchical and complex designs, identification of the appropriate level for tests and full reporting of outcomes
☐	☒	Estimates of effect sizes (e.g. Cohen's *d*, Pearson's *r*), indicating how they were calculated

Our web collection on statistics for biologists contains articles on many of the points above.

Software and code

Policy information about availability of computer code

Data collection	Analysis code and software used to analyze micropost sensors and run prototype hardware is available on github (https://github.com/lhting/Trauma_Study_GUI). This MATLAB software interfaces with the machine hardware (heater, camera, pump) and automatically runs simultaneous analysis on the image sensor data.
Data analysis	Analysis code and software used to analyze micropost sensors and run prototype hardware is available on github (https://github.com/lhting/Trauma_Study_GUI). This MATLAB software reads images put out by the camera, and analyzes the positions of the micropost position relative to the block, and outputs force and other measurements to memory.

For manuscripts utilizing custom algorithms or software that are central to the research but not yet described in published literature, software must be made available to editors/reviewers. We strongly encourage code deposition in a community repository (e.g. GitHub). See the Nature Research guidelines for submitting code & software for further information.

Data

Policy information about availability of data

All manuscripts must include a data availability statement. This statement should provide the following information, where applicable:

- Accession codes, unique identifiers, or web links for publicly available datasets
- A list of figures that have associated raw data
- A description of any restrictions on data availability

All relevant data are available from the corresponding author on reasonable request.

Field-specific reporting

Please select the one below that is the best fit for your research. If you are not sure, read the appropriate sections before making your selection.

☒ Life sciences ☐ Behavioural & social sciences ☐ Ecological, evolutionary & environmental sciences

For a reference copy of the document with all sections, see nature.com/documents/nr-reporting-summary-flat.pdf

Life sciences study design

All studies must disclose on these points even when the disclosure is negative.

Sample size	For the trauma study total N=110 trauma subjects and N=10 healthy controls
Data exclusions	No data were excluded from the trauma study.
Replication	Measurements taken from blood collected from trauma patients could not be replicated due to ethical limitations on volume of blood drawn from eash subject.
Randomization	Not relevant, these were prospective observational studies.
Blinding	Not relevant, these were not randomized clinical studies

Reporting for specific materials, systems and methods

We require information from authors about some types of materials, experimental systems and methods used in many studies. Here, indicate whether each material, system or method listed is relevant to your study. If you are not sure if a list item applies to your research, read the appropriate section before selecting a response.

Materials & experimental systems

n/a	Involved in the study	
☒	☐	Antibodies
☒	☐	Eukaryotic cell lines
☒	☐	Palaeontology
☒	☐	Animals and other organisms
☐	☒	Human research participants
☒	☐	Clinical data

Methods

n/a	Involved in the study	
☒	☐	ChIP-seq
☐	☒	Flow cytometry
☒	☐	MRI-based neuroimaging

Human research participants

Policy information about studies involving human research participants

Population characteristics	Trauma subjects were predominantly male and >80% were bluntly-injured, which is typical for trauma patients.
Recruitment	Subjects for the trauma study were enrolled from the local population of Seattle, WA USA if they were adult (>18 years old), not pregnant or incarcerated, presented directly from the place of injury, and had received no more than three units of transfused blood products prior to a blood draw in the Emergency Department.
Ethics oversight	The trauma study protocol was approved by the University of Washington Human Subjects Division.

Note that full information on the approval of the study protocol must also be provided in the manuscript.

Flow Cytometry

Plots

Confirm that:

☒ The axis labels state the marker and fluorochrome used (e.g. CD4-FITC).

☒ The axis scales are clearly visible. Include numbers along axes only for bottom left plot of group (a 'group' is an analysis of identical markers).

☒ All plots are contour plots with outliers or pseudocolor plots.

☒ A numerical value for number of cells or percentage (with statistics) is provided.

Methodology

Sample preparation	Human blood samples that were passed through a microfluidic device
Instrument	BD LSR II flow cytometer
Software	FlowJo v10
Cell population abundance	Whole blood samples were not sorted prior to testing.
Gating strategy	For each sample, in an aliquot stained with an isotype control antibody with the same fluorophore as its corresponding marker (IgG-PE for CD62p-PE and IgM-FITC for PAC-1-FITC), a gate was drawn that was positive for less than 4% of platelets. This defined the limit of nonspecific binding. The number of platelets positive for nonspecific binding was recorded for each sample. Applying the same gates set in the isotype control aliquot, the number of platelets positive for the CD62p or PAC-1 marker was determined in an aliquot stained with those markers. The final number of platelets with CD62p- or PAC-1-specific binding was calculated by subtracting the number of positive platelets in the control aliquot from the number of positive platelets in the marker aliquot.

☒ Tick this box to confirm that a figure exemplifying the gating strategy is provided in the Supplementary Information.
